# Association of surgeon and hospital volume with short-term outcomes after robot-assisted radical prostatectomy: Nationwide, population-based study

**DOI:** 10.1371/journal.pone.0253081

**Published:** 2021-06-17

**Authors:** Rebecka Arnsrud Godtman, Erik Persson, Walter Cazzaniga, Fredrik Sandin, Stefan Carlsson, Göran Ahlgren, Eva Johansson, David Robinsson, Jonas Hugosson, Pär Stattin

**Affiliations:** 1 Department of Urology, Institute of Clinical Sciences, Sahlgrenska Academy at University of Göteborg, Sahlgrenska University Hospital, Göteborg, Sweden; 2 Regional Cancer Center Uppsala Örebro, Uppsala, Sweden; 3 Division of Experimental Oncology/Unit of Urology URI, IRCCS Ospedale San Raffaele, University Vita-Salute San Raffaele, Milan, Italy; 4 Department of Surgical Sciences, Uppsala University, Uppsala, Sweden; 5 Division of Urology, Karolinska University Hospital, Stockholm, Sweden; 6 Department of Molecular Medicine and Surgery (MMK), Karolinska Institutet, Stockholm, Sweden; 7 Department of Urology, Skåne University Hospital, Lund University, Lund, Sweden; 8 Department of Urology, Högland Hospital, Eksjö, Sweden; University of Sydney, AUSTRALIA

## Abstract

**Background and objective:**

Few studies have investigated the association between surgical volume and outcome of robot-assisted radical prostatectomy (RARP) in an unselected cohort. We sought to investigate the association between surgical volume with peri-operative and short-term outcomes in a nation-wide, population-based study group.

**Methods:**

9,810 RARP’s registered in the National Prostate Cancer Register of Sweden (2015–2018) were included. Associations between outcome and volume were analyzed with multivariable logistic regression including age, PSA-density, number of positive biopsy cores, cT stage, Gleason score, and extent of lymph node dissection.

**Results:**

Surgeons and hospitals in the highest volume group compared to lowest group had shorter operative time; surgeon (OR 9.20, 95% CI 7.11–11.91), hospital (OR 2.16, 95% CI 1.53–3.06), less blood loss; surgeon (OR 2.58. 95% CI 2.07–3.21) hospital (no difference), more often nerve sparing intention; surgeon (OR 2.89, 95% CI 2.34–3.57), hospital (OR 2.02, 95% CI 1.66–2.44), negative margins; surgeon (OR 1.90, 95% CI 1.54–2.35), hospital (OR 1.28, 95% CI 1.07–1.53). There was wide range in outcome between hospitals and surgeons with similar volume that remained after adjustment.

**Conclusions:**

High surgeon and hospital volume were associated with better outcomes. The range in outcome was wide in all volume groups, which indicates that factors besides volume are of importance. Registration of surgical performance is essential for quality control and improvement.

## Introduction

Already 40 years ago, Luft and colleagues described an association between high hospital surgical volume and lower mortality and suggested a centralization of certain surgical procedures [1]. Since then there has been many publications in support of an association between high surgical volume and better outcome [2, 3].

Radical prostatectomy (RP) is no exception and numerous publications have reported an association between high volume and good outcomes including risk of complications, readmission, positive surgical margin, incontinence, risk of disease recurrence, mortality and costs [4, 5]. These reports have stimulated initiatives aiming at centralization of RP. For example, the NICE guidelines state that at least 150 robot assisted radical prostatectomy (RARP)/year should be performed in a hospital in order to ensure cost-effectiveness [6] and in Germany, a minimum of 50 RP’s are required in order for a clinic to be a certified as a “prostate cancer center” [7]. Parallel to this process there has been a movement in the opposite direction with wide dissemination of robotic technology so even some small hospitals in Sweden have invested in surgical robots. A decentralization of RP´s has also been reported from e.g. Germany where RP’s are performed at an increasing number of hospitals [7].

Compared to the large number of reports on outcome after open retropubic RP, there are few studies on the association between volume and outcome for RARP. To date these reports have been either large population-based studies with limited possibilities to adjust for case mix or reports with comprehensive data from specialized tertial referral centers [4, 8].

The aim of this study was to investigate the association between surgeon and hospital volume with perioperative and short-term outcomes in a nationwide population-based cohort of men.

## Materials and methods

The National Prostate Cancer Register (NPCR) of Sweden captures 98% of all newly diagnosed prostate cancer cases in Sweden [9]. NPCR collects information on cancer characteristics, work-up, primary treatment and since 2015, also comprehensive information on RP and radical radiotherapy. A detailed description of the RP form (PiS: Prostatectomy in Sweden form) has been published [10]. In summary, the PiS form contain pre-, peri-, and postoperative variables and exists in a short version with 60 variables and a more comprehensive version with 83 variables. Each reporting department decides which form to use. The variable list for the two PiS forms is available in Swedish at: https://www.cancercentrum.se/samverkan/cancerdiagnoser/prostata/kvalitetsregister/dokument/. NPCR registers the identity of each individual surgeon by use of a code and the code key is kept at each department. A majority of RP surgeons in Sweden performs surgeries at one hospital but some surgeons perform RP´s at several hospitals and are thus registered with multiple codes. Data on re-admission and comorbidity were obtained by linkage to the Patient Registry in Prostate Cancer data Base Sweden (PCBaSe RAPID 2018) [11]. Charlson comorbidity index (CCI) was calculated as previously described [12, 13]. NPCR/PCBaSe RAPID has been approved by the Research Ethics Board Uppsala (Dnr 2017–263). The board waived the need for consent. In Sweden, cancer registers use an opt-out design for consent, meaning that individuals are informed but no written consent is collected. If an individual does not want to participate, he will not be registered in NPCR/PCBaSe.

**Fig 1:**
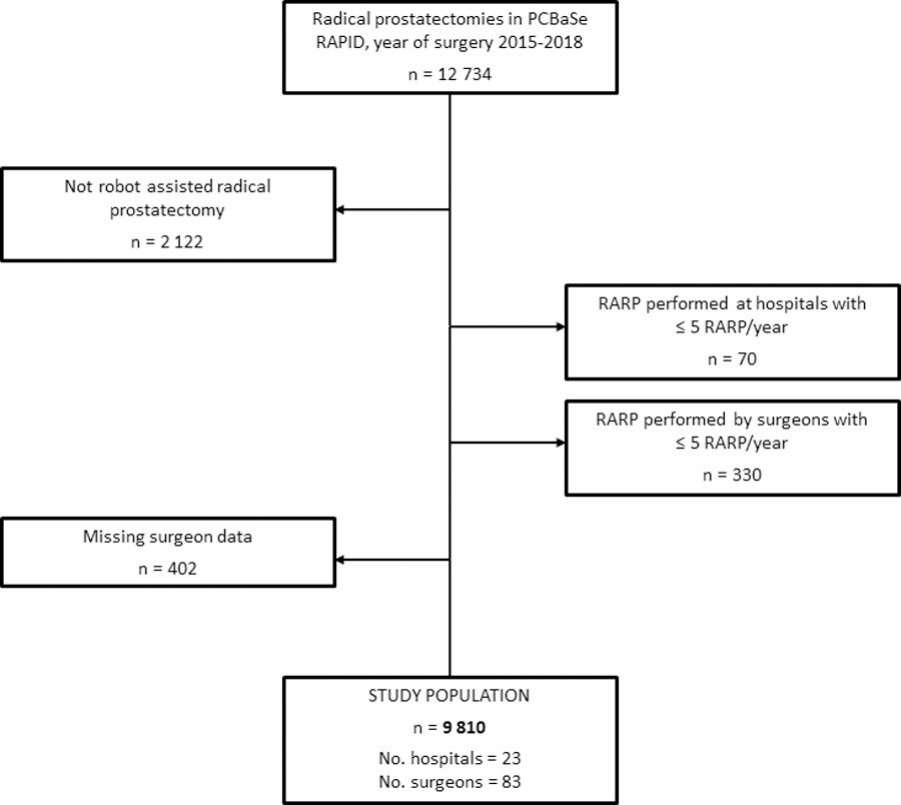
Flow chart of the study population in Prostate Cancer data Base Sweden (PCBaSe).

The present study included all RARP´s registered in NPCR performed between 1^st^ Jan 2015 and 31^st^ Dec 2018. RARP´s performed after an initial period of active surveillance were not included (Fig 1). Hospitals and surgeons who had registered ≤5 RARP/year were excluded.

### Statistical analysis

Cut-off values for surgeon and hospital volume were based on the Swedish national guidelines for prostate cancer that recommend that a RP surgeon should perform at least 25 RP’s per year and that there should be at least two surgeons at each department who perform this number of RP’s [14]. Multiples of these numbers were used to define cut-off values for each volume group: very low (surgeon <13/ hospital <50), low (surgeon 13-25/hospital 50–100), intermediate (surgeon 25-50/hospital 100–150), high (50-75/ hospital 150–200) and very high (surgeon ≥75/ hospital ≥200). The mean number of RARP’s per year was calculated by dividing the accumulated number of RARP’s per surgeon and hospital with the study period of 4 years. The grouping of hospital volume was performed before the exclusion of RARP’s with missing data on surgeon identity.

The Multiple Imputation Chained Equations (MICE) method [15] was used to impute missing data on PSA, prostate volume, number of positive biopsy cores, cT stage, Gleason score and extent of lymph node dissection. The following additional variables were included to improve predictions: region, patient age at surgery, year of surgery, total mm of cancer in biopsy cores, number of biopsies with cancer, vital status, and time from surgery to death or 31^st^ of December 2018, whichever event came first. Data were imputed 20 times and results from the models fitted to each dataset were pooled using Rubin’s rules [16].

Outcome variables were dichotomized; thresholds for short operative time and low blood loss were based on the median. Univariable logistic regression was used to calculate odds ratios (OR) for the outcomes. In the multivariable logistic regression we included age at RARP, PSA, prostate volume, PSA density, number of positive biopsy cores, cT stage, Gleason score, and extent of lymph node dissection. Surgeon and hospital volume were analysed in two separate models. The multivariable logistic regression models were subsequently used to construct covariate-adjusted funnel plots using the method proposed by Spiegelhalter [17].

All statistical analyses were performed in R Statistical Software (Version 3.6.1; Foundation for Statistical Computing, Vienna, Austria).

## Results

10,612 men in NPCR underwent a RARP between 1^st^ Jan 2015 and 31^st^ Dec 2018, 802 men were excluded leaving a final study population of 9,810 men (Fig 1). Of the 10,612 men, 9,868 were also reported in the National Patient Registry, while 740 men were registered in NPCR only, and 430 men registered in the Patient Register only. 74% (7252/9810) of all procedures were performed by surgeons who performed >25 RARP/year and 58% (5734/9810) of all RARP were performed at high or very high volume hospitals. Table 1 demonstrates clinical characteristics of the study population according to surgeon volume (hospital volume S1 Table). There was no clear association between case mix and surgeon or hospital volume. There was a wide range in the proportion of lymph node dissection with the highest proportion in RARP’s performed by high volume surgeons and at very high volume hospital. Very high volume surgeons and hospitals had the highest completeness of registration of data.

**Table 1:** Baseline characteristics in Prostate Cancer data Base Sweden (PCBaSe) according to surgical volume; number of robot assisted radical prostatectomies (RARP) performed by a single surgeon.

		Very low volume;		Low volume;		Intermediate volume;		High volume;		Very high volume;		Total	
		<13 RARP/year		13–25 RARP/year		25–50 RARP/year		50–75 RARP/year		>75 RARP/year			
		*(N = 604)*		*(N = 1954)*		*(N = 3552)*		*(N = 2123)*		*(N = 1577)*		*(N = 9810)*	
**Patient age at RP**													
	Median (IQR)	66	(61–70)	66	(61–70)	66	(61–70)	66	(60–70)	65	(60–70)	66	(60–70)
	<65	244	(40.4)	791	(40.5)	1544	(43.5)	938	(44.2)	738	(46.8)	4255	(43.4)
	65–75	345	(57.1)	1110	(56.8)	1951	(54.9)	1130	(53.2)	782	(49.6)	5318	(54.2)
	>75	15	(2.5)	53	(2.7)	57	(1.6)	55	(2.6)	57	(3.6)	237	(2.4)
**Charlson Comorbidity Index, No. (%)**													
	0	486	(80.5)	1503	(76.9)	2807	(79.0)	1688	(79.5)	1255	(79.6)	7739	(78.9)
	1	65	(10.8)	250	(12.8)	389	(11.0)	237	(11.2)	181	(11.5)	1122	(11.4)
	2+	53	(8.8)	201	(10.3)	356	(10.0)	198	(9.3)	141	(8.9)	949	(9.7)
**PSA, No. (%)**													
	Median (IQR)	7	(5–10.3)	6.7	(4.7–10)	6.8	(4.7–10.9)	6.8	(4.6–10.4)	6.9	(4.5–11)	6.8	(4.7–10.4)
	<3 ng/ml	25	(4.1)	84	(4.3)	158	(4.4)	86	(4.1)	94	(6.0)	447	(4.6)
	3–10 ng/ml	422	(69.9)	1382	(70.7)	2455	(69.1)	1480	(69.7)	1058	(67.1)	6797	(69.3)
	10.1–20 ng/ml	129	(21.4)	344	(17.6)	662	(18.6)	391	(18.4)	297	(18.8)	1823	(18.6)
	>20 ng/ml	26	(4.3)	108	(5.5)	256	(7.2)	142	(6.7)	127	(8.1)	659	(6.7)
	Missing	2	(0.3)	36	(1.8)	21	(0.6)	24	(1.1)	1	(0.1)	84	(0.9)
**Prostate volume, No. (%)**													
	Median (IQR)	36	(28–46)	36	(28–48)	36	(28–50)	36	(28–48)	36	(28–47)	36	(28–48)
	<30 ml	173	(28.6)	511	(26.2)	974	(27.4)	582	(27.4)	464	(29.4)	2704	(27.6)
	30–60 ml	360	(59.6)	1120	(57.3)	2072	(58.3)	1206	(56.8)	908	(57.6)	5666	(57.8)
	61–90 ml	47	(7.8)	152	(7.8)	309	(8.7)	186	(8.8)	132	(8.4)	826	(8.4)
	>90 ml	8	(1.3)	53	(2.7)	77	(2.2)	44	(2.1)	52	(3.3)	234	(2.4)
	Missing	16	(2.6)	118	(6.0)	120	(3.4)	105	(4.9)	21	(1.3)	380	(3.9)
**PSA density, No. (%)**													
	<0.1	55	(9.1)	256	(13.1)	432	(12.2)	280	(13.2)	217	(13.8)	1240	(12.6)
	0.1–0.2	256	(42.4)	775	(39.7)	1463	(41.2)	841	(39.6)	645	(40.9)	3980	(40.6)
	>0.2	277	(45.9)	799	(40.9)	1535	(43.2)	889	(41.9)	693	(43.9)	4193	(42.7)
	Missing	16	(2.6)	124	(6.3)	122	(3.4)	113	(5.3)	22	(1.4)	397	(4.0)
**Number of biopsies, No. (%)**													
	≤6	16	(2.6)	48	(2.5)	72	(2.0)	35	(1.6)	40	(2.5)	211	(2.2)
	7–10	235	(38.9)	553	(28.3)	1100	(31.0)	1032	(48.6)	440	(27.9)	3360	(34.3)
	11–12	251	(41.6)	939	(48.1)	1681	(47.3)	676	(31.8)	760	(48.2)	4307	(43.9)
	>12	22	(3.6)	94	(4.8)	193	(5.4)	108	(5.1)	78	(4.9)	495	(5.0)
	Missing	80	(13.2)	320	(16.4)	506	(14.2)	272	(12.8)	259	(16.4)	1437	(14.6)
**Number of positive biopsies, No. (%)**													
	≤2	129	(21.4)	505	(25.8)	801	(22.6)	468	(22.0)	320	(20.3)	2223	(22.7)
	3–4	163	(27.0)	447	(22.9)	929	(26.2)	580	(27.3)	390	(24.7)	2509	(25.6)
	5–6	139	(23.0)	362	(18.5)	726	(20.4)	458	(21.6)	315	(20.0)	2000	(20.4)
	>6	90	(14.9)	316	(16.2)	574	(16.2)	339	(16.0)	289	(18.3)	1608	(16.4)
	Missing	83	(13.7)	324	(16.6)	522	(14.7)	278	(13.1)	263	(16.7)	1470	(15.0)
**Total mm of cancer in biopsies**													
	Median (IQR)	13.5	(7–25.7)	12.1	(5–24.5)	13.5	(6–26.9)	13	(5.6–26)	14.7	(6.7–27.9)	13.4	(6–26)
**Clinical T stage, No. (%)**													
	T1a/T1b	5	(0.8)	13	(0.7)	14	(0.4)	14	(0.7)	11	(0.7)	57	(0.6)
	T1c	331	(54.8)	1116	(57.1)	1950	(54.9)	1255	(59.1)	896	(56.8)	5548	(56.6)
	T2	236	(39.1)	719	(36.8)	1352	(38.1)	721	(34.0)	587	(37.2)	3615	(36.9)
	T3	25	(4.1)	34	(1.7)	140	(3.9)	94	(4.4)	60	(3.8)	353	(3.6)
	T4	0	(0.0)	0	(0.0)	2	(0.1)	1	(0.0)	0	(0.0)	3	(0.0)
	TX/Missing	7	(1.2)	72	(3.7)	94	(2.6)	38	(1.8)	23	(1.5)	234	(2.4)
**Clinical N stage, No. (%)**													
	N0	271	(44.9)	906	(46.4)	1690	(47.6)	977	(46.0)	702	(44.5)	4546	(46.3)
	N1	10	(1.7)	32	(1.6)	82	(2.3)	51	(2.4)	15	(1.0)	190	(1.9)
	NX	323	(53.5)	1016	(52.0)	1780	(50.1)	1095	(51.6)	860	(54.5)	5074	(51.7)
**Clinical M stage, No. (%)**													
	M0	600	(99.3)	1932	(98.9)	3507	(98.7)	2108	(99.3)	1560	(98.9)	9707	(99.0)
	M1	0	(0.0)	8	(0.4)	14	(0.4)	6	(0.3)	1	(0.1)	29	(0.3)
	MX	4	(0.7)	14	(0.7)	31	(0.9)	9	(0.4)	16	(1.0)	74	(0.8)
**Gleason score, No. (%)**													
	Gleason score 6	127	(21.0)	473	(24.2)	776	(21.8)	485	(22.8)	369	(23.4)	2230	(22.7)
	Gleason score 7 (3+4)	256	(42.4)	853	(43.7)	1546	(43.5)	861	(40.6)	610	(38.7)	4126	(42.1)
	Gleason score 7 (4+3)	148	(24.5)	400	(20.5)	787	(22.2)	502	(23.6)	345	(21.9)	2182	(22.2)
	Gleason score 8	37	(6.1)	105	(5.4)	237	(6.7)	163	(7.7)	147	(9.3)	689	(7.0)
	Gleason score 9–10	23	(3.8)	85	(4.4)	158	(4.4)	94	(4.4)	84	(5.3)	444	(4.5)
	Missing	13	(2.2)	38	(1.9)	48	(1.4)	18	(0.8)	22	(1.4)	139	(1.4)
**Risk category, No. (%)**													
	Very low risk	11	(1.8)	53	(2.7)	55	(1.5)	57	(2.7)	39	(2.5)	215	(2.2)
	Low risk	87	(14.4)	286	(14.6)	480	(13.5)	289	(13.6)	245	(15.5)	1387	(14.1)
	Intermediate	397	(65.7)	1226	(62.7)	2226	(62.7)	1305	(61.5)	911	(57.8)	6065	(61.8)
	High risk	64	(10.6)	240	(12.3)	478	(13.5)	287	(13.5)	287	(18.2)	1356	(13.8)
	Locally advanced	21	(3.5)	25	(1.3)	108	(3.0)	81	(3.8)	49	(3.1)	284	(2.9)
	Regionally metastatic	14	(2.3)	44	(2.3)	106	(3.0)	62	(2.9)	29	(1.8)	255	(2.6)
	Distant metastasis	1	(0.2)	8	(0.4)	25	(0.7)	10	(0.5)	5	(0.3)	49	(0.5)
	Missing	9	(1.5)	72	(3.7)	74	(2.1)	32	(1.5)	12	(0.8)	199	(2.0)
**Surgical margin status, No. (%)**													
	Negative	389	(64.4)	1340	(68.6)	2227	(62.7)	1392	(65.6)	1214	(77.0)	6562	(66.9)
	Positive	188	(31.1)	552	(28.2)	1181	(33.2)	663	(31.2)	345	(21.9)	2929	(29.9)
	Unclear	17	(2.8)	39	(2.0)	127	(3.6)	53	(2.5)	12	(0.8)	248	(2.5)
	Missing	10	(1.7)	23	(1.2)	17	(0.5)	15	(0.7)	6	(0.4)	71	(0.7)
**Operation time (min)**													
	Median (IQR)	180	(149–220)	158	(130–196)	153	(125–188)	152	(120–188)	135	(114.5–165.5)	150	(120–188)
	≤100	8	(1.3)	55	(2.8)	281	(7.9)	164	(7.7)	231	(14.6)	739	(7.5)
	101–150	113	(18.7)	478	(24.5)	1200	(33.8)	610	(28.7)	798	(50.6)	3199	(32.6)
	151–200	135	(22.4)	356	(18.2)	969	(27.3)	492	(23.2)	256	(16.2)	2208	(22.5)
	>200	143	(23.7)	259	(13.3)	597	(16.8)	297	(14.0)	170	(10.8)	1466	(14.9)
	Missing	205	(33.9)	806	(41.2)	505	(14.2)	560	(26.4)	122	(7.7)	2198	(22.4)
**Blood loss (ml)**													
	Median (IQR)	100	(75–200)	150	(100–227.5)	100	(75–200)	100	(50–200)	100	(50–150)	100	(50–200)
	<100	129	(21.4)	324	(16.6)	832	(23.4)	597	(28.1)	748	(47.4)	2630	(26.8)
	100–249	241	(39.9)	651	(33.3)	1704	(48.0)	777	(36.6)	587	(37.2)	3960	(40.4)
	250–499	67	(11.1)	270	(13.8)	480	(13.5)	186	(8.8)	125	(7.9)	1128	(11.5)
	500–999	20	(3.3)	45	(2.3)	120	(3.4)	60	(2.8)	45	(2.9)	290	(3.0)
	≥1000	2	(0.3)	10	(0.5)	12	(0.3)	9	(0.4)	4	(0.3)	37	(0.4)
	Missing	145	(24.0)	654	(33.5)	404	(11.4)	494	(23.3)	68	(4.3)	1765	(18.0)
**Blood transfusion**													
	No	328	(54.3)	1022	(52.3)	2768	(77.9)	1180	(55.6)	1531	(97.1)	6829	(69.6)
	Yes	2	(0.3)	16	(0.8)	38	(1.1)	23	(1.1)	15	(1.0)	94	(1.0)
	Missing	274	(45.4)	916	(46.9)	746	(21.0)	920	(43.3)	31	(2.0)	2887	(29.4)
**Number of transfusion units**													
	≤2	1	(50.0)	9	(56.2)	24	(63.2)	11	(47.8)	12	(80.0)	57	(60.6)
	2–6	0	(0.0)	5	(31.2)	10	(26.3)	9	(39.1)	3	(20.0)	27	(28.7)
	7–10	1	(50.0)	2	(12.5)	2	(5.3)	3	(13.0)	0	(0.0)	8	(8.5)
	>10	0	(0.0)	0	(0.0)	2	(5.3)	0	(0.0)	0	(0.0)	2	(2.1)
**Lymph node dissection, No. (%)**													
	Not performed	528	(87.4)	1656	(84.7)	2910	(81.9)	1575	(74.2)	1204	(76.3)	7873	(80.3)
	Limited	3	(0.5)	7	(0.4)	19	(0.5)	30	(1.4)	14	(0.9)	73	(0.7)
	Extended	73	(12.1)	291	(14.9)	622	(17.5)	518	(24.4)	358	(22.7)	1862	(19.0)
	Missing	0	(0.0)	0	(0.0)	1	(0.0)	0	(0.0)	1	(0.1)	2	(0.0)
**Nerve sparing procedure, No. (%)**													
	Yes	297	(49.2)	1132	(57.9)	2189	(61.6)	1532	(72.2)	1083	(68.7)	6233	(63.5)
	No	305	(50.5)	816	(41.8)	1347	(37.9)	589	(27.7)	483	(30.6)	3540	(36.1)
	Missing	2	(0.3)	6	(0.3)	16	(0.5)	2	(0.1)	11	(0.7)	37	(0.4)
**Duration of stay (days)**													
	1	269	(44.5)	1061	(54.3)	1718	(48.4)	891	(42.0)	853	(54.1)	4792	(48.8)
	2–3	280	(46.4)	739	(37.8)	1609	(45.3)	996	(46.9)	351	(22.3)	3975	(40.5)
	4–7	38	(6.3)	96	(4.9)	168	(4.7)	107	(5.0)	29	(1.8)	438	(4.5)
	>7	9	(1.5)	19	(1.0)	44	(1.2)	28	(1.3)	14	(0.9)	114	(1.2)
	Missing	8	(1.3)	39	(2.0)	13	(0.4)	101	(4.8)	330	(20.9)	491	(5.0)
**Upgrading, No. (%)**													
	No	469	(77.6)	1459	(74.7)	2804	(78.9)	1649	(77.7)	1289	(81.7)	7670	(78.2)
	Yes	120	(19.9)	456	(23.3)	702	(19.8)	459	(21.6)	263	(16.7)	2000	(20.4)
	Missing	15	(2.5)	39	(2.0)	46	(1.3)	15	(0.7)	25	(1.6)	140	(1.4)
**Upstaging, No. (%)**													
	No	394	(65.2)	1199	(61.4)	2253	(63.4)	1314	(61.9)	964	(61.1)	6124	(62.4)
	Yes	189	(31.3)	660	(33.8)	1208	(34.0)	764	(36.0)	590	(37.4)	3411	(34.8)
	Missing	21	(3.5)	95	(4.9)	91	(2.6)	45	(2.1)	23	(1.5)	275	(2.8)
**Readmission rate. (%)**													
	No	557	(92.2)	1787	(91.5)	3281	(92.4)	1979	(93.2)	1420	(90.0)	9024	(92.0)
	Yes	47	(7.8)	167	(8.5)	271	(7.6)	144	(6.8)	157	(10.0)	786	(8.0)
**Duration of readmission (days)**													
	≤3	32	(68.1)	113	(67.7)	178	(65.7)	91	(63.2)	92	(58.6)	506	(64.4)
	4–7	10	(21.3)	34	(20.4)	65	(24.0)	35	(24.3)	41	(26.1)	185	(23.5)
	8–15	2	(4.3)	17	(10.2)	25	(9.2)	13	(9.0)	14	(8.9)	71	(9.0)
	>15	3	(6.4)	3	(1.8)	3	(1.1)	5	(3.5)	10	(6.4)	24	(3.1)

Limits for volume groups are shown as mean number of RARP/year performed by each surgeon. CCI is calculated at diagnosis.

Table 2 demonstrates OR for the factors that were included as adjustments in the multivariable analysis of surgeon volume (hospital volume S2 Table). Prostate volume and lymph node dissection was associated with long operative time and high blood loss. There were fewer negative surgical margins with increasing PSA, PSA density, number of positive cores, higher T stage, and extended lymph node dissection. Nerve sparing intention was less common with increasing patient age, comorbidity, PSA, prostate volume, number of positive cores, cT stage, and Gleason score. Extended lymph node dissection was negatively associated with all outcomes except nerve sparing intention. Results were virtually identical when we analyzed these factors in a complete case analysis.

**Table 2:** Odds ratios (OR) and 95% confidence intervals (CI) of the covariates for respective outcome according to surgical volume by single surgeon.

		Short operative time		Low blood loss		Nerve sparing		Negative margins		No readmission	
		OR	(95% CI)	OR	(95% CI)	OR	(95% CI)	OR	(95% CI)	OR	(95% CI)
**Patient age at RP**											
	<65	1.00	(ref.)	1.00	(ref.)	1.00	(ref.)	1.00	(ref.)	1.00	(ref.)
	65–75	1.06	(0.96–1.18)	1.19	(1.08–1.31)	0.47	(0.43–0.52)	0.87	(0.79–0.95)	0.88	(0.75–1.04)
	>75	1.37	(0.98–1.92)	1.75	(1.28–2.41)	0.24	(0.18–0.32)	0.92	(0.69–1.24)	0.66	(0.43–1.01)
**Charlson Comorbidity Index**											
	0	1.00	(ref.)	1.00	(ref.)	1.00	(ref.)	1.00	(ref.)	1.00	(ref.)
	1	0.96	(0.82–1.12)	1.04	(0.90–1.21)	0.83	(0.72–0.95)	0.98	(0.85–1.12)	0.70	(0.57–0.87)
	2+	1.21	(1.02–1.43)	1.26	(1.08–1.47)	0.80	(0.69–0.93)	0.95	(0.82–1.10)	0.80	(0.63–1.02)
**PSA**											
	<3 ng/ml	1.00	(ref.)	1.00	(ref.)	1.00	(ref.)	1.00	(ref.)	1.00	(ref.)
	3–10 ng/ml	1.22	(0.94–1.59)	0.92	(0.72–1.17)	0.88	(0.68–1.12)	0.89	(0.69–1.14)	1.09	(0.74–1.59)
	10.1–20 ng/ml	1.14	(0.84–1.56)	0.81	(0.61–1.07)	0.62	(0.46–0.82)	0.65	(0.49–0.86)	0.99	(0.64–1.53)
	>20 ng/ml	1.11	(0.77–1.60)	0.82	(0.59–1.13)	0.33	(0.23–0.45)	0.41	(0.30–0.56)	0.98	(0.60–1.59)
**Prostate volyme**											
	<30 ml	1.00	(ref.)	1.00	(ref.)	1.00	(ref.)	1.00	(ref.)	1.00	(ref.)
	30–60 ml	0.80	(0.71–0.91)	0.76	(0.67–0.85)	0.90	(0.80–1.01)	1.01	(0.90–1.12)	0.90	(0.75–1.09)
	61–90 ml	0.54	(0.43–0.67)	0.53	(0.44–0.65)	0.80	(0.66–0.98)	1.18	(0.97–1.43)	0.93	(0.68–1.28)
	>90 ml	0.33	(0.23–0.48)	0.36	(0.26–0.50)	0.66	(0.48–0.91)	1.14	(0.82–1.57)	0.81	(0.50–1.32)
**PSA density**											
	<0.1	1.00	(ref.)	1.00	(ref.)	1.00	(ref.)	1.00	(ref.)	1.00	(ref.)
	0.1–0.2	1.01	(0.86–1.18)	0.97	(0.84–1.13)	1.09	(0.94–1.26)	0.81	(0.70–0.94)	1.05	(0.83–1.33)
	>0.2	1.10	(0.91–1.33)	1.21	(1.02–1.45)	0.87	(0.74–1.03)	0.62	(0.52–0.73)	1.03	(0.79–1.35)
**Positive biopsies**											
	≤2	1.00	(ref.)	1.00	(ref.)	1.00	(ref.)	1.00	(ref.)	1.00	(ref.)
	3–4	1.04	(0.90–1.19)	1.00	(0.88–1.14)	1.00	(0.88–1.15)	1.08	(0.95–1.22)	0.96	(0.77–1.20)
	5–6	1.14	(0.98–1.34)	0.99	(0.85–1.14)	0.86	(0.75–0.99)	0.92	(0.81–1.05)	0.97	(0.77–1.22)
	>6	1.11	(0.94–1.32)	0.99	(0.85–1.15)	0.39	(0.33–0.45)	0.76	(0.66–0.88)	0.92	(0.73–1.17)
**Clinical T stage**											
	T1	1.00	(ref.)	1.00	(ref.)	1.00	(ref.)	1.00	(ref.)	1.00	(ref.)
	T2	1.12	(1.01–1.25)	1.09	(0.99–1.21)	0.74	(0.67–0.82)	1.00	(0.91–1.10)	1.02	(0.87–1.20)
	T3/T4	0.98	(0.72–1.32)	0.89	(0.69–1.14)	0.29	(0.22–0.38)	0.75	(0.59–0.95)	0.92	(0.64–1.30)
**Gleason score**											
	Gleason score 6	1.00	(ref.)	1.00	(ref.)	1.00	(ref.)	1.00	(ref.)	1.00	(ref.)
	Gleason score 7 (3+4)	0.91	(0.80–1.04)	0.99	(0.87–1.11)	0.84	(0.74–0.95)	1.19	(1.06–1.34)	1.11	(0.90–1.36)
	Gleason score 7 (4+3)	0.98	(0.84–1.15)	1.16	(1.01–1.34)	0.72	(0.63–0.83)	0.98	(0.86–1.12)	0.99	(0.78–1.24)
	Gleason score 8	1.24	(0.98–1.59)	0.99	(0.80–1.22)	0.53	(0.43–0.65)	1.32	(1.07–1.62)	1.03	(0.75–1.41)
	Gleason score 9–10	1.26	(0.92–1.73)	1.01	(0.79–1.30)	0.22	(0.17–0.29)	1.03	(0.81–1.31)	0.88	(0.62–1.25)
**Lymph node dissection**											
	Not performed	1.00	(ref.)	1.00	(ref.)	1.00	(ref.)	1.00	(ref.)	1.00	(ref.)
	Limited	0.37	(0.21–0.64)	0.61	(0.36–1.04)	1.07	(0.63–1.82)	1.55	(0.89–2.70)	1.00	(0.40–2.51)
	Extended	0.09	(0.07–0.11)	0.77	(0.67–0.89)	0.94	(0.81–1.08)	0.79	(0.69–0.90)	0.51	(0.41–0.62)

**Fig 2:**
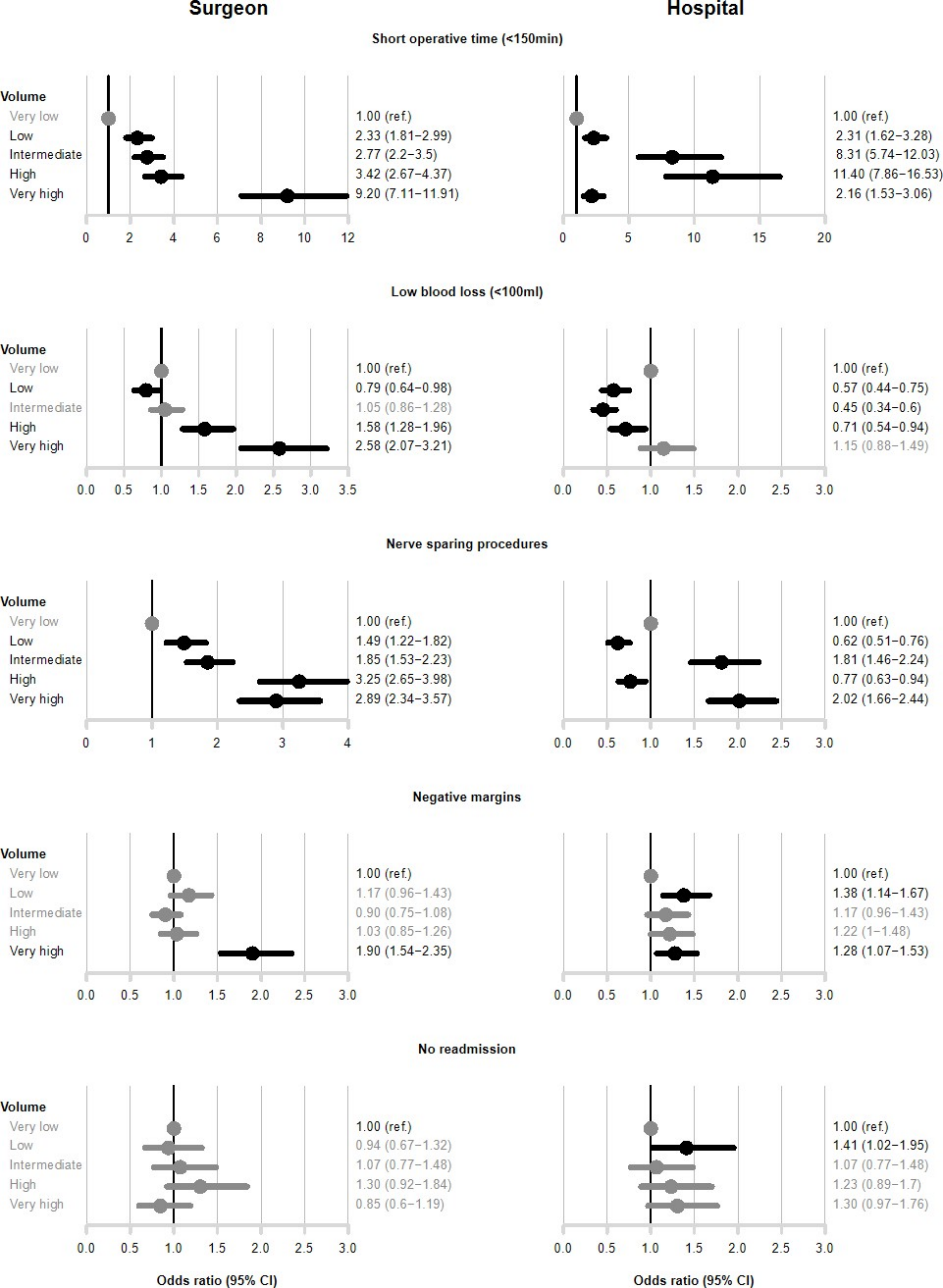
Odds ratios (OR) and 95% confidence intervals (CI) of outcome according to surgical volume by single surgeon and hospital.

Fig 2 shows outcomes from multivariable analyses adjusted for clinical characteristics including surgeon and hospital volume. Surgeons and hospitals in the highest volume group had, compared to the lowest volume group, shorter operative time, surgeon OR 9.20 (95% CI 7.11–11.91), hospital OR 2.16 (95% CI 1.53–3.06). Blood loss was smaller in RARP’s performed by surgeons in the highest volume group compared to the lowest volume group (OR 2.58, 95% CI 2.07–3.21). There was an U-shaped, negative association between hospital volume and blood loss. Surgeons and hospitals in the highest volume groups had higher odds of nerve sparing intent compared to very low volume surgeons (OR 2.89, 95% CI 2.34–3.57) and hospitals (OR 2.2, 95% CI 1.66–2.44). Negative surgical margins were more common in procedures performed by the highest volume surgeons compared to the lowest volume surgeons (OR 1.90, 95% CI 1.54–2.35). There was no clear association between hospital volume and negative surgical margins. There was no statistically significant association between volume and readmission with the exception of low volume hospitals which had higher odds (1.41, 95% CI 1.02–1.95) for no readmission compared to very low volume hospitals.

**Fig 3:**
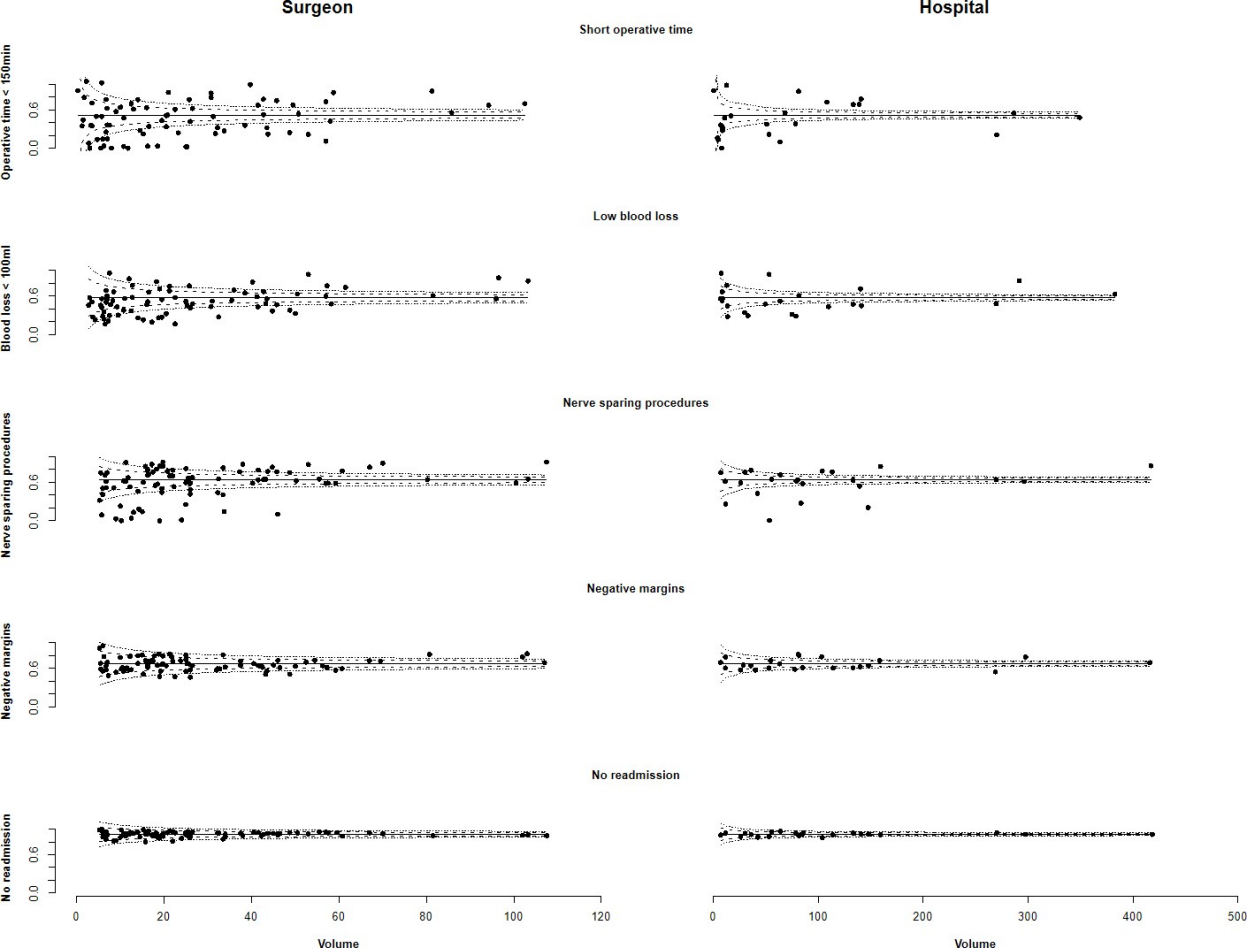
Adjusted proportions of RARP with outcome according to surgical volume by single surgeon and hospital. Mean proportion and 95% and 99.9% CI for the whole data set also displayed. Mean number of RARP/year performed by each surgeon and in each hospital is shown on the x-axis.

Fig 3 shows adjusted funnel plots for the five outcomes. For individual surgeons, there was a wide range in the proportion of procedures with a short operative time and low blood loss across the range of volume. All very high volume surgeons were at or above the median value for short operative time and low blood loss but the range was wide also in this group and there were a few ‘positive outliers’. For hospital volume, the widest range in operative time and blood loss was observed among very low volume hospitals. There was a wide range in proportion of nerve sparing procedure for all surgeon volumes. The proportion of nerve sparing procedures was highest among very high volume hospitals. All very high volume surgeons were above the median value for high proportion of negative margins. For hospital volume, the widest range was observed in very high volume hospitals. Compared to the other outcomes, there was a smaller range in the proportion of men with no readmission for volume according to both surgeon and hospital.

## Discussion

In this nation-wide, population-based register study on robot-assisted radical prostatectomy (RARP), highest vs lowest volume surgeons had shorter operative time, less blood loss, more often performed nerve sparing intent and had more negative surgical margins also when adjusting for case mix and potential confounders. For hospital volume of RARP, associations between volume and perioperative outcomes were weaker. Some low volume surgeons and low volume hospitals had better results than some surgeons and hospitals with higher volume, however the low number of observations increased the risk of chance findings in the former groups. There were also high volume surgeons and hospitals who performed below average.

### Strength and limitations

The strengths of this study include the nation-wide, population-based study group with a virtually complete capture of all men in Sweden who underwent RARP at all types of hospitals. In contrast to many studies based on administrative registries, we had access to detailed patient and cancer characteristics enabling us to adjust for potential confounders in our analyses. Another strength is inclusion of both surgeon and hospital volume. Exclusion of surgeons and hospitals with <5 RARP/year could have introduced a bias. We argue for the opposite, that including these observations would have led to erroneous estimates since it is likely that many of these registrations were of poor quality. With this threshold, we avoided erroneous registration of RARP at hospitals where there is no surgical robot or registration of RARP performed by visiting high volume surgeon at a small hospital. The proportion of missing data was small for all variables in the model except for number of positive biopsy cores. We applied multiple imputation to handle missing data, however, results were virtually identical when we analyzed these factors in a complete case analysis. Limitations of our study include lack of data on functional and long-term oncological outcomes and surgical experience for individual surgeons prior to the study period. Given this lack of data we had to assume that all surgeons practiced during the entire study period, which would “falsely” lower the average number of RARP’s/year for some surgeons.

A systematic review on the association between volume and outcome after RP reported 49 original publications of which 11 studies examined both surgeon and hospital volume [4]. High surgeon and hospital RP volume were associated with better outcome, however, the association varied between outcomes. For example, cost and mortality were more dependent on institutional factors whereas incontinence and length of hospital stay were more related to individual surgeon [4].

We found a clear dose-response association between surgeon volume and short operative time and low blood loss, which could not be observed for hospital volume, indicating that these outcomes are more dependent on the individual surgeon’s technique than on hospital factors. Other studies have reported similar associations [18, 19]. Surgeons and hospitals in the highest volume groups had higher odds of a nerve sparing intent compared to very low volume surgeons and hospitals. However, nerve sparing intent was based on the surgeon´s self assessment and is not an outcome and in order to investigate the validity of this variable, data on functional outcomes are needed.

We found that the highest volume surgeons had almost two-fold higher odds of negative surgical margins vs. the lowest volume surgeons. Surgical margin status in organ confined prostate cancer is an important measure of surgical quality and positive surgical margins are associated with increased risk of biochemical recurrence and secondary treatment [20]. Most previous studies have reported a higher proportion of negative margins with increasing RP volume [21–23]. For example, in a recent study based on the American National Cancer Database, there was a higher proportion of negative margins in high volume hospitals [22]. Besides surgical technique, margin status is also affected by T stage, Gleason, quality of the pathology assessment etc, which could explain the lack of association with hospital volume and the wide range between hospitals in the same volume group.

We found no association between readmission and volume which is in line with a previous study from PCBaSe [24] but in contrast to a register-based US study [22].

The type of volume-outcomes association differed between outcomes. For example, for operative time and blood loss, we found a dose-response association with improved outcome with increased volume but for negative surgical margins, there seemed to be a threshold effect where only the very-high volume group had significantly better results. One potential explanation could be that the learning curve to reach improved oncological outcome with RARP is longer than the learning curve for operative time and blood loss.

We speculate that surgeon volume is the driver in the volume-outcome association. Compared to many previous studies, the association between hospital volume and perioperative outcomes was weak in our study [4], likely due to the fact there were very high volume hospitals with many low to intermediate volume surgeons and lower volume hospitals with high/very high volume surgeons.

We observed a wide range in outcomes also for surgeons and hospitals within the same volume group. However, for most of the outcomes, variation within a volume group was wider with decreasing volumes. For example, the largest variation in operative time was seen for surgeons and hospitals in the lowest volume groups.

The proportion of men who received a nerve sparing procedure spanned from less than 10% to close to 100% between very low volume surgeons and from less than 30% to more than 90% between high volume hospitals. Similarily, the proportion of negative surgical margins in very high volume surgeons varied from <70% to >80%. For readmission, the variation was smaller in all volume groups.

In these comparisons within a volume group, there should be limited influence by case-mix suggesting that there are other factors that affect outcome. Random effect can probably explain some variation, especially in the lower volume groups. There are numerous reports describing heterogeneity between surgeons, including those with high volumes [25–29]. In a recent population-based, Swedish study, significant variation in functional and oncological outcomes was seen, also for experiences surgeons. Even tough surgeons’ experience and annual volume could explain some of the observed heterogeneity in outcomes, most of the heterogeneity remained after adjusting for these factors. Individual surgeon skill and talent probably explain some variation. Some surgeons are more skilled than others are, but to what extent these skills can be improved by training is unknown and probably differs between individuals [29].

Our results do not answer whether prostate cancer surgery can or should be centralized but rather emphasize the need to register and report surgical performance in order to ensure quality control and quality improvement. When outcomes are registered, best practice can be identified and disseminated. Continuous registration and feedback from registers such as the NPCR can help raise the minimum quality standards for surgeons and hospitals regardless of volume. We argue that quality registration should be compulsory since. quality assurance of RP has been shown to improve outcome. For example, in a quality assurance program at the Pelvic cancer surgical center in London, UK complication rate fell from 13% to 7% and urinary continence 3 months after surgery increased from 57% to 67% [30].

In 2018, NPCR introduced electronic forms for patient reported outcome measures (ePROM) for men undergoing RP and radical radiotherapy. Patients are asked to fill in electronic questionnaires before treatment, and at 3 and 12 months after treatment. The results are immediately available online at the secured Information Network for Cancer Care (INCA) platform where comparisons between regions, hospitals, and individual surgeons within the same hospital can be performed. Capture was limited in 2018, so there is not enough data as of yet to investigate the association between volume and PROM.

## Conclusions

In this nation-wide, population-based register study, we found strong associations between high surgeon volume and good perioperative and short-term outcomes whereas for hospitals, association between volume and outcome was weaker. There was a wide range in outcome between hospitals and surgeons with similar surgical volume indicating that factors other than volume are important. These findings highlight the need to register and report surgical performance to facilitate quality control and improvement. Future studies in NPCR will include functional results based on ePROM.

## Supporting information

S1 Table Baseline characteristics of men in Prostate Cancer data Base Sweden (PCBaSe) according to hospital volume; mean number of robot assisted radical prostatectomies (RARP)/year performed in a hospital. Limits for volume groups are shown as mean number of RARP/year performed in a hospital. CCI is calculated at diagnosis.
(DOCX)

S2 Table Odds ratios (OR) and 95% confidence intervals (CI) of the covariates for respective outcome according to surgical volume in a hospital. (DOCX)

S1 File List of the hospitals included. (DOCX)
