# Supplementary material for: Association of surgeon and hospital volume with short-term outcomes after robot-assisted radical prostatectomy: Nationwide, population-based study
Source: PLoS One. 2021 Jun 17;16(6):e0253081. doi: 10.1371/journal.pone.0253081 (PMC8211177; doi:10.1371/journal.pone.0253081)
Supplement: S1 Table — Limits for volume groups are shown as mean number of RARP/year performed in a hospital. CCI is calculated at diagnosis. (DOCX) [file pone.0253081.s001.docx]

| **S1 Table. Baseline characteristics in Prostate Cancer data Base Sweden (PCBaSe) according to hospital volume; number of robot assisted radical prostatectomies (RARP) performed in a hospital.** | | | | | | | | | | | | | |
| --- | --- | --- | --- | --- | --- | --- | --- | --- | --- | --- | --- | --- | --- |
|  |  | **Very low volume;** | | **Low volume;** | | **Intermediate volume;** | | **High volume;** | | **Very high volume;** | | **Total** | |
|  |  | **<50 RARP/year** | | **50-100 RARP/year** | | **100-150 RARP/year** | | **150-200 RARP/year** | | **>200 RARP/year** | |  |  |
|  |  | ***(N = 660)*** | | ***(N = 2012)*** | | ***(N = 1404)*** | | ***(N = 1790)*** | | ***(N = 3944)*** | | ***(N = 9810)*** | |
| **Patient age at RP** | |  |  |  |  |  |  |  |  |  |  |  |  |
|  | Median (IQR) | 66 | (61-69) | 66 | (61-70) | 66 | (61-70) | 65 | (60-69) | 66 | (60-70) | 66 | (60-70) |
|  | <65 | 278 | (42.1) | 827 | (41.1) | 554 | (39.5) | 850 | (47.5) | 1746 | (44.3) | 4255 | (43.4) |
|  | 65-75 | 373 | (56.5) | 1125 | (55.9) | 823 | (58.6) | 908 | (50.7) | 2089 | (53.0) | 5318 | (54.2) |
|  | >75 | 9 | (1.4) | 60 | (3.0) | 27 | (1.9) | 32 | (1.8) | 109 | (2.8) | 237 | (2.4) |
| **Charlson Comorbidity Index, No. (%)** | |  |  |  |  |  |  |  |  |  |  |  |  |
|  | 0 | 540 | (81.8) | 1587 | (78.9) | 1123 | (80.0) | 1401 | (78.3) | 3088 | (78.3) | 7739 | (78.9) |
|  | 1 | 70 | (10.6) | 232 | (11.5) | 157 | (11.2) | 197 | (11.0) | 466 | (11.8) | 1122 | (11.4) |
|  | 2+ | 50 | (7.6) | 193 | (9.6) | 124 | (8.8) | 192 | (10.7) | 390 | (9.9) | 949 | (9.7) |
| **PSA, No. (%)** | |  |  |  |  |  |  |  |  |  |  |  |  |
|  | Median (IQR) | 7.1 | (5.1-11) | 6.7 | (4.7-10) | 6.7 | (4.8-10.5) | 6.6 | (4.5-10) | 6.9 | (4.7-11) | 6.8 | (4.7-10.4) |
|  | <3 ng/ml | 15 | (2.3) | 69 | (3.4) | 52 | (3.7) | 72 | (4.0) | 239 | (6.1) | 447 | (4.6) |
|  | 3-10 ng/ml | 463 | (70.2) | 1441 | (71.6) | 975 | (69.4) | 1265 | (70.7) | 2653 | (67.3) | 6797 | (69.3) |
|  | 10.1-20 ng/ml | 140 | (21.2) | 376 | (18.7) | 265 | (18.9) | 306 | (17.1) | 736 | (18.7) | 1823 | (18.6) |
|  | >20 ng/ml | 38 | (5.8) | 98 | (4.9) | 89 | (6.3) | 123 | (6.9) | 311 | (7.9) | 659 | (6.7) |
|  | Missing | 4 | (0.6) | 28 | (1.4) | 23 | (1.6) | 24 | (1.3) | 5 | (0.1) | 84 | (0.9) |
| **Prostate volume, No. (%)** | |  |  |  |  |  |  |  |  |  |  |  |  |
|  | Median (IQR) | 37 | (30-50) | 36 | (28-47) | 36 | (28-50) | 36 | (28-48) | 36 | (28-48) | 36 | (28-48) |
|  | <30 ml | 147 | (22.3) | 550 | (27.3) | 369 | (26.3) | 501 | (28.0) | 1137 | (28.8) | 2704 | (27.6) |
|  | 30-60 ml | 402 | (60.9) | 1159 | (57.6) | 781 | (55.6) | 1037 | (57.9) | 2287 | (58.0) | 5666 | (57.8) |
|  | 61-90 ml | 54 | (8.2) | 162 | (8.1) | 136 | (9.7) | 125 | (7.0) | 349 | (8.8) | 826 | (8.4) |
|  | >90 ml | 10 | (1.5) | 46 | (2.3) | 28 | (2.0) | 42 | (2.3) | 108 | (2.7) | 234 | (2.4) |
|  | Missing | 47 | (7.1) | 95 | (4.7) | 90 | (6.4) | 85 | (4.7) | 63 | (1.6) | 380 | (3.9) |
| **PSA density, No. (%)** | |  |  |  |  |  |  |  |  |  |  |  |  |
|  | <0.1 | 66 | (10.0) | 221 | (11.0) | 159 | (11.3) | 242 | (13.5) | 552 | (14.0) | 1240 | (12.6) |
|  | 0.1-0.2 | 265 | (40.2) | 882 | (43.8) | 561 | (40.0) | 734 | (41.0) | 1538 | (39.0) | 3980 | (40.6) |
|  | >0.2 | 282 | (42.7) | 809 | (40.2) | 591 | (42.1) | 724 | (40.4) | 1787 | (45.3) | 4193 | (42.7) |
|  | Missing | 47 | (7.1) | 100 | (5.0) | 93 | (6.6) | 90 | (5.0) | 67 | (1.7) | 397 | (4.0) |
| **Number of biopsies, No. (%)** | |  |  |  |  |  |  |  |  |  |  |  |  |
|  | ≤6 | 28 | (4.2) | 42 | (2.1) | 24 | (1.7) | 27 | (1.5) | 90 | (2.3) | 211 | (2.2) |
|  | 7-10 | 282 | (42.7) | 926 | (46.0) | 408 | (29.1) | 595 | (33.2) | 1149 | (29.1) | 3360 | (34.3) |
|  | 11-12 | 256 | (38.8) | 670 | (33.3) | 688 | (49.0) | 836 | (46.7) | 1857 | (47.1) | 4307 | (43.9) |
|  | >12 | 22 | (3.3) | 73 | (3.6) | 90 | (6.4) | 115 | (6.4) | 195 | (4.9) | 495 | (5.0) |
|  | Missing | 72 | (10.9) | 301 | (15.0) | 194 | (13.8) | 217 | (12.1) | 653 | (16.6) | 1437 | (14.6) |
| **Number of positive biopsies, No. (%)** | |  |  |  |  |  |  |  |  |  |  |  |  |
|  | ≤2 | 105 | (15.9) | 561 | (27.9) | 324 | (23.1) | 417 | (23.3) | 816 | (20.7) | 2223 | (22.7) |
|  | 3-4 | 190 | (28.8) | 511 | (25.4) | 361 | (25.7) | 452 | (25.3) | 995 | (25.2) | 2509 | (25.6) |
|  | 5-6 | 177 | (26.8) | 389 | (19.3) | 280 | (19.9) | 370 | (20.7) | 784 | (19.9) | 2000 | (20.4) |
|  | >6 | 115 | (17.4) | 246 | (12.2) | 244 | (17.4) | 312 | (17.4) | 691 | (17.5) | 1608 | (16.4) |
|  | Missing | 73 | (11.1) | 305 | (15.2) | 195 | (13.9) | 239 | (13.4) | 658 | (16.7) | 1470 | (15.0) |
| **Total mm of cancer in biopsies** | |  |  |  |  |  |  |  |  |  |  |  |  |
|  | Median (IQR) | 17 | (8.5-30) | 11 | (5-22) | 13 | (6-25) | 12.3 | (5-26) | 14.5 | (7-27.8) | 13.4 | (6-26) |
| **Clinical T stage, No. (%)** | |  |  |  |  |  |  |  |  |  |  |  |  |
|  | T1a/T1b | 2 | (0.3) | 15 | (0.7) | 11 | (0.8) | 10 | (0.6) | 19 | (0.5) | 57 | (0.6) |
|  | T1c | 360 | (54.5) | 1289 | (64.1) | 809 | (57.6) | 1007 | (56.3) | 2083 | (52.8) | 5548 | (56.6) |
|  | T2 | 249 | (37.7) | 628 | (31.2) | 502 | (35.8) | 655 | (36.6) | 1581 | (40.1) | 3615 | (36.9) |
|  | T3 | 28 | (4.2) | 27 | (1.3) | 38 | (2.7) | 90 | (5.0) | 170 | (4.3) | 353 | (3.6) |
|  | T4 | 0 | (0.0) | 1 | (0.0) | 1 | (0.1) | 0 | (0.0) | 1 | (0.0) | 3 | (0.0) |
|  | TX/Missing | 21 | (3.2) | 52 | (2.6) | 43 | (3.1) | 28 | (1.6) | 90 | (2.3) | 234 | (2.4) |
| **Clinical N stage, No. (%)** | |  |  |  |  |  |  |  |  |  |  |  |  |
|  | N0 | 335 | (50.8) | 981 | (48.8) | 601 | (42.8) | 638 | (35.6) | 1991 | (50.5) | 4546 | (46.3) |
|  | N1 | 12 | (1.8) | 34 | (1.7) | 6 | (0.4) | 17 | (0.9) | 121 | (3.1) | 190 | (1.9) |
|  | NX | 313 | (47.4) | 997 | (49.6) | 797 | (56.8) | 1135 | (63.4) | 1832 | (46.5) | 5074 | (51.7) |
| **Clinical M stage, No. (%)** | |  |  |  |  |  |  |  |  |  |  |  |  |
|  | M0 | 657 | (99.5) | 1995 | (99.2) | 1382 | (98.4) | 1783 | (99.6) | 3890 | (98.6) | 9707 | (99.0) |
|  | M1 | 0 | (0.0) | 4 | (0.2) | 8 | (0.6) | 1 | (0.1) | 16 | (0.4) | 29 | (0.3) |
|  | MX | 3 | (0.5) | 13 | (0.6) | 14 | (1.0) | 6 | (0.3) | 38 | (1.0) | 74 | (0.8) |
| **Gleason score, No. (%)** | |  |  |  |  |  |  |  |  |  |  |  |  |
|  | Gleason score 6 | 124 | (18.8) | 513 | (25.5) | 328 | (23.4) | 434 | (24.2) | 831 | (21.1) | 2230 | (22.7) |
|  | Gleason score 7 (3+4) | 247 | (37.4) | 863 | (42.9) | 592 | (42.2) | 799 | (44.6) | 1625 | (41.2) | 4126 | (42.1) |
|  | Gleason score 7 (4+3) | 200 | (30.3) | 427 | (21.2) | 318 | (22.6) | 378 | (21.1) | 859 | (21.8) | 2182 | (22.2) |
|  | Gleason score 8 | 41 | (6.2) | 114 | (5.7) | 87 | (6.2) | 100 | (5.6) | 347 | (8.8) | 689 | (7.0) |
|  | Gleason score 9-10 | 30 | (4.5) | 63 | (3.1) | 64 | (4.6) | 63 | (3.5) | 224 | (5.7) | 444 | (4.5) |
|  | Missing | 18 | (2.7) | 32 | (1.6) | 15 | (1.1) | 16 | (0.9) | 58 | (1.5) | 139 | (1.4) |
| **Risk category, No. (%)** | |  |  |  |  |  |  |  |  |  |  |  |  |
|  | Very low risk | 11 | (1.7) | 69 | (3.4) | 27 | (1.9) | 51 | (2.8) | 57 | (1.4) | 215 | (2.2) |
|  | Low risk | 74 | (11.2) | 299 | (14.9) | 187 | (13.3) | 272 | (15.2) | 555 | (14.1) | 1387 | (14.1) |
|  | Intermediate | 434 | (65.8) | 1293 | (64.3) | 888 | (63.2) | 1106 | (61.8) | 2344 | (59.4) | 6065 | (61.8) |
|  | High risk | 80 | (12.1) | 225 | (11.2) | 200 | (14.2) | 217 | (12.1) | 634 | (16.1) | 1356 | (13.8) |
|  | Locally advanced | 22 | (3.3) | 22 | (1.1) | 34 | (2.4) | 81 | (4.5) | 125 | (3.2) | 284 | (2.9) |
|  | Regionally metastatic | 18 | (2.7) | 43 | (2.1) | 17 | (1.2) | 31 | (1.7) | 146 | (3.7) | 255 | (2.6) |
|  | Distant metastasis | 1 | (0.2) | 6 | (0.3) | 8 | (0.6) | 4 | (0.2) | 30 | (0.8) | 49 | (0.5) |
|  | Missing | 20 | (3.0) | 55 | (2.7) | 43 | (3.1) | 28 | (1.6) | 53 | (1.3) | 199 | (2.0) |
|  |  |  |  |  |  |  |  |  |  |  |  |  |  |
| **Surgical margin status, No. (%)** | |  |  |  |  |  |  |  |  |  |  |  |  |
|  | Negative | 402 | (60.9) | 1400 | (69.6) | 929 | (66.2) | 1200 | (67.0) | 2631 | (66.7) | 6562 | (66.9) |
|  | Positive | 203 | (30.8) | 527 | (26.2) | 436 | (31.1) | 543 | (30.3) | 1220 | (30.9) | 2929 | (29.9) |
|  | Unclear | 36 | (5.5) | 57 | (2.8) | 36 | (2.6) | 41 | (2.3) | 78 | (2.0) | 248 | (2.5) |
|  | Missing | 19 | (2.9) | 28 | (1.4) | 3 | (0.2) | 6 | (0.3) | 15 | (0.4) | 71 | (0.7) |
| **Operation time (min)** | |  |  |  |  |  |  |  |  |  |  |  |  |
|  | Median (IQR) | 174.5 | (150-208.2) | 155 | (120-194.5) | 130 | (110-152) | 125 | (110-150) | 166 | (138-205) | 150 | (120-188) |
|  | ≤100 | 2 | (0.3) | 213 | (10.6) | 155 | (11.0) | 230 | (12.8) | 139 | (3.5) | 739 | (7.5) |
|  | 101-150 | 49 | (7.4) | 560 | (27.8) | 616 | (43.9) | 671 | (37.5) | 1303 | (33.0) | 3199 | (32.6) |
|  | 151-200 | 72 | (10.9) | 480 | (23.9) | 215 | (15.3) | 226 | (12.6) | 1215 | (30.8) | 2208 | (22.5) |
|  | >200 | 51 | (7.7) | 362 | (18.0) | 47 | (3.3) | 42 | (2.3) | 964 | (24.4) | 1466 | (14.9) |
|  | Missing | 486 | (73.6) | 397 | (19.7) | 371 | (26.4) | 621 | (34.7) | 323 | (8.2) | 2198 | (22.4) |
| **Blood loss (ml)** | |  |  |  |  |  |  |  |  |  |  |  |  |
|  | Median (IQR) | 100 | (50-200) | 125 | (75-200) | 150 | (100-250) | 100 | (50-200) | 100 | (50-180) | 100 | (50-200) |
|  | <100 | 103 | (15.6) | 446 | (22.2) | 200 | (14.2) | 412 | (23.0) | 1469 | (37.2) | 2630 | (26.8) |
|  | 100-249 | 122 | (18.5) | 822 | (40.9) | 602 | (42.9) | 647 | (36.1) | 1767 | (44.8) | 3960 | (40.4) |
|  | 250-499 | 35 | (5.3) | 287 | (14.3) | 223 | (15.9) | 176 | (9.8) | 407 | (10.3) | 1128 | (11.5) |
|  | 500-999 | 4 | (0.6) | 91 | (4.5) | 57 | (4.1) | 19 | (1.1) | 119 | (3.0) | 290 | (3.0) |
|  | ≥1000 | 1 | (0.2) | 12 | (0.6) | 9 | (0.6) | 3 | (0.2) | 12 | (0.3) | 37 | (0.4) |
|  | Missing | 395 | (59.8) | 354 | (17.6) | 313 | (22.3) | 533 | (29.8) | 170 | (4.3) | 1765 | (18.0) |
| **Blood transfusion** | |  |  |  |  |  |  |  |  |  |  |  |  |
|  | No | 89 | (13.5) | 1556 | (77.3) | 941 | (67.0) | 687 | (38.4) | 3556 | (90.2) | 6829 | (69.6) |
|  | Yes | 1 | (0.2) | 21 | (1.0) | 27 | (1.9) | 2 | (0.1) | 43 | (1.1) | 94 | (1.0) |
|  | Missing | 570 | (86.4) | 435 | (21.6) | 436 | (31.1) | 1101 | (61.5) | 345 | (8.7) | 2887 | (29.4) |
| **Number of transfusion units** | |  |  |  |  |  |  |  |  |  |  |  |  |
|  | ≤2 | 0 | (0.0) | 15 | (71.4) | 13 | (48.1) | 1 | (50.0) | 28 | (65.1) | 57 | (60.6) |
|  | 2-6 | 0 | (0.0) | 4 | (19.0) | 10 | (37.0) | 1 | (50.0) | 12 | (27.9) | 27 | (28.7) |
|  | 7-10 | 1 | (100.0) | 2 | (9.5) | 2 | (7.4) | 0 | (0.0) | 3 | (7.0) | 8 | (8.5) |
|  | >10 | 0 | (0.0) | 0 | (0.0) | 2 | (7.4) | 0 | (0.0) | 0 | (0.0) | 2 | (2.1) |
| **Lymph node dissection, No. (%)** | |  |  |  |  |  |  |  |  |  |  |  |  |
|  | Not performed | 596 | (90.3) | 1793 | (89.1) | 1220 | (86.9) | 1471 | (82.2) | 2793 | (70.8) | 7873 | (80.3) |
|  | Limited | 9 | (1.4) | 9 | (0.4) | 11 | (0.8) | 31 | (1.7) | 13 | (0.3) | 73 | (0.7) |
|  | Extended | 54 | (8.2) | 210 | (10.4) | 173 | (12.3) | 288 | (16.1) | 1137 | (28.8) | 1862 | (19.0) |
|  | Missing | 1 | (0.2) | 0 | (0.0) | 0 | (0.0) | 0 | (0.0) | 1 | (0.0) | 2 | (0.0) |
| **Nerve sparing procedure, No. (%)** | |  |  |  |  |  |  |  |  |  |  |  |  |
|  | Yes | 392 | (59.4) | 1062 | (52.8) | 997 | (71.0) | 996 | (55.6) | 2786 | (70.6) | 6233 | (63.5) |
|  | No | 264 | (40.0) | 939 | (46.7) | 403 | (28.7) | 789 | (44.1) | 1145 | (29.0) | 3540 | (36.1) |
|  | Missing | 4 | (0.6) | 11 | (0.5) | 4 | (0.3) | 5 | (0.3) | 13 | (0.3) | 37 | (0.4) |
| **Duration of stay (days)** | |  |  |  |  |  |  |  |  |  |  |  |  |
|  | 1 | 253 | (38.3) | 873 | (43.4) | 967 | (68.9) | 924 | (51.6) | 1775 | (45.0) | 4792 | (48.8) |
|  | 2-3 | 347 | (52.6) | 632 | (31.4) | 332 | (23.6) | 701 | (39.2) | 1963 | (49.8) | 3975 | (40.5) |
|  | 4-7 | 33 | (5.0) | 127 | (6.3) | 70 | (5.0) | 53 | (3.0) | 155 | (3.9) | 438 | (4.5) |
|  | >7 | 11 | (1.7) | 24 | (1.2) | 29 | (2.1) | 12 | (0.7) | 38 | (1.0) | 114 | (1.2) |
|  | Missing | 16 | (2.4) | 356 | (17.7) | 6 | (0.4) | 100 | (5.6) | 13 | (0.3) | 491 | (5.0) |
| **Upgrading, No. (%)** | |  |  |  |  |  |  |  |  |  |  |  |  |
|  | No | 517 | (78.3) | 1469 | (73.0) | 1105 | (78.7) | 1404 | (78.4) | 3175 | (80.5) | 7670 | (78.2) |
|  | Yes | 126 | (19.1) | 510 | (25.3) | 288 | (20.5) | 375 | (20.9) | 701 | (17.8) | 2000 | (20.4) |
|  | Missing | 17 | (2.6) | 33 | (1.6) | 11 | (0.8) | 11 | (0.6) | 68 | (1.7) | 140 | (1.4) |
| **Upstaging, No. (%)** | |  |  |  |  |  |  |  |  |  |  |  |  |
|  | No | 399 | (60.5) | 1245 | (61.9) | 927 | (66.0) | 1168 | (65.3) | 2385 | (60.5) | 6124 | (62.4) |
|  | Yes | 216 | (32.7) | 698 | (34.7) | 432 | (30.8) | 582 | (32.5) | 1483 | (37.6) | 3411 | (34.8) |
|  | Missing | 45 | (6.8) | 69 | (3.4) | 45 | (3.2) | 40 | (2.2) | 76 | (1.9) | 275 | (2.8) |
| **Readmission rate. (%)** | |  |  |  |  |  |  |  |  |  |  |  |  |
|  | No | 601 | (91.1) | 1878 | (93.3) | 1281 | (91.2) | 1650 | (92.2) | 3614 | (91.6) | 9024 | (92.0) |
|  | Yes | 59 | (8.9) | 134 | (6.7) | 123 | (8.8) | 140 | (7.8) | 330 | (8.4) | 786 | (8.0) |
| **Duration of readmission (days)** | |  |  |  |  |  |  |  |  |  |  |  |  |
|  | ≤3 | 40 | (67.8) | 83 | (61.9) | 83 | (67.5) | 96 | (68.6) | 204 | (61.8) | 506 | (64.4) |
|  | 4-7 | 13 | (22.0) | 35 | (26.1) | 26 | (21.1) | 31 | (22.1) | 80 | (24.2) | 185 | (23.5) |
|  | 8-15 | 4 | (6.8) | 9 | (6.7) | 14 | (11.4) | 12 | (8.6) | 32 | (9.7) | 71 | (9.0) |
|  | >15 | 2 | (3.4) | 7 | (5.2) | 0 | (0.0) | 1 | (0.7) | 14 | (4.2) | 24 | (3.1) |
| **Limits for volume groups are shown as mean number of RARP/year performed in a hospital. CCI is calculated at diagnosis.** | | | | | | | |  |  |  |  |  |  |
|  |  |  |  |  |  |  |  |  |  |  |  |  |  |
|  |  |  |  |  |  |  |  |  |  |  |  |  |  |
|  |  |  |  |  |  |  |  |  |  |  |  |  |  |
|  |  |  |  |  |  |  |  |  |  |  |  |  |  |
|  |  |  |  |  |  |  |  |  |  |  |  |  |  |
|  |  |  |  |  |  |  |  |  |  |  |  |  |  |
