# Supplementary material for: Association of surgeon and hospital volume with short-term outcomes after robot-assisted radical prostatectomy: Nationwide, population-based study
Source: PLoS One. 2021 Jun 17;16(6):e0253081. doi: 10.1371/journal.pone.0253081 (PMC8211177; doi:10.1371/journal.pone.0253081)
Supplement: S2 Table — (DOCX) [file pone.0253081.s002.docx]

| **S2 Table. Odds ratios (OR) and 95% confidence intervals (CI) of the covariates for respective outcome according to surgical volume in a hospital** | | | | | | | | | | | | |  |  |
| --- | --- | --- | --- | --- | --- | --- | --- | --- | --- | --- | --- | --- | --- | --- |
|  |  |  | **Short operative time** | | **Low blood loss** | | **Nerve sparing** | | **Negative margins** | | **No readmission** | |  |  |
|  |  |  | **OR** | **(95% CI)** | **OR** | **(95% CI)** | **OR** | **(95% CI)** | **OR** | **(95% CI)** | **OR** | **(95% CI)** |  |  |
| **Patient age at RP** | |  |  |  |  |  |  |  |  |  |  |  |  |  |
|  | <65 |  | 1.00 | (ref.) | 1.00 | (ref.) | 1.00 | (ref.) | 1.00 | (ref.) | 1.00 | (ref.) |  |  |
|  | 65-75 |  | 0.94 | (0.85-1.05) | 1.20 | (1.09-1.32) | 0.46 | (0.42-0.51) | 0.86 | (0.78-0.94) | 0.89 | (0.76-1.04) |  |  |
|  | >75 |  | 1.60 | (1.14-2.25) | 1.83 | (1.33-2.51) | 0.24 | (0.18-0.33) | 0.97 | (0.72-1.29) | 0.64 | (0.42-0.97) |  |  |
| **Charlson Comorbidity Index** | | | |  |  |  |  |  |  |  |  |  |  |  |
|  | 0 |  | 1.00 | (ref.) | 1.00 | (ref.) | 1.00 | (ref.) | 1.00 | (ref.) | 1.00 | (ref.) |  |  |
|  | 1 |  | 0.97 | (0.82-1.14) | 1.02 | (0.89-1.18) | 0.81 | (0.70-0.94) | 0.98 | (0.86-1.13) | 0.70 | (0.56-0.86) |  |  |
|  | 2+ |  | 1.12 | (0.94-1.33) | 1.23 | (1.05-1.44) | 0.80 | (0.69-0.93) | 0.94 | (0.82-1.09) | 0.79 | (0.63-1.01) |  |  |
| **PSA** | |  |  |  |  |  |  |  |  |  |  |  |  |  |
|  | <3 ng/ml |  | 1.00 | (ref.) | 1.00 | (ref.) | 1.00 | (ref.) | 1.00 | (ref.) | 1.00 | (ref.) |  |  |
|  | 3-10 ng/ml |  | 1.00 | (0.76-1.30) | 0.99 | (0.77-1.25) | 1.00 | (0.78-1.29) | 0.87 | (0.68-1.12) | 1.14 | (0.78-1.67) |  |  |
|  | 10.1-20 ng/ml |  | 0.87 | (0.64-1.19) | 0.91 | (0.68-1.20) | 0.72 | (0.54-0.96) | 0.64 | (0.48-0.84) | 1.04 | (0.67-1.62) |  |  |
|  | >20 ng/ml |  | 0.78 | (0.54-1.13) | 0.96 | (0.69-1.32) | 0.38 | (0.27-0.53) | 0.41 | (0.30-0.56) | 1.03 | (0.63-1.68) |  |  |
| **Prostate volyme** | |  |  |  |  |  |  |  |  |  |  |  |  |  |
|  | <30 ml |  | 1.00 | (ref.) | 1.00 | (ref.) | 1.00 | (ref.) | 1.00 | (ref.) | 1.00 | (ref.) |  |  |
|  | 30-60 ml |  | 0.80 | (0.70-0.91) | 0.75 | (0.67-0.84) | 0.88 | (0.78-0.99) | 1.01 | (0.91-1.12) | 0.90 | (0.75-1.09) |  |  |
|  | 61-90 ml |  | 0.52 | (0.41-0.65) | 0.53 | (0.43-0.64) | 0.74 | (0.61-0.91) | 1.18 | (0.97-1.43) | 0.93 | (0.68-1.28) |  |  |
|  | >90 ml |  | 0.37 | (0.25-0.54) | 0.35 | (0.25-0.49) | 0.61 | (0.44-0.85) | 1.18 | (0.85-1.62) | 0.78 | (0.48-1.27) |  |  |
| **PSA density** | |  |  |  |  |  |  |  |  |  |  |  |  |  |
|  | <0.1 |  | 1.00 | (ref.) | 1.00 | (ref.) | 1.00 | (ref.) | 1.00 | (ref.) | 1.00 | (ref.) |  |  |
|  | 0.1-0.2 |  | 1.03 | (0.87-1.22) | 0.97 | (0.83-1.12) | 1.04 | (0.90-1.20) | 0.81 | (0.70-0.94) | 1.03 | (0.82-1.31) |  |  |
|  | >0.2 |  | 1.13 | (0.93-1.37) | 1.16 | (0.98-1.38) | 0.78 | (0.66-0.93) | 0.62 | (0.53-0.73) | 1.01 | (0.77-1.32) |  |  |
| **Positive biopsies** | |  |  |  |  |  |  |  |  |  |  |  |  |  |
|  | ≤2 |  | 1.00 | (ref.) | 1.00 | (ref.) | 1.00 | (ref.) | 1.00 | (ref.) | 1.00 | (ref.) |  |  |
|  | 3-4 |  | 1.10 | (0.95-1.27) | 1.01 | (0.89-1.15) | 0.99 | (0.86-1.13) | 1.09 | (0.96-1.23) | 0.98 | (0.78-1.22) |  |  |
|  | 5-6 |  | 1.18 | (1.01-1.39) | 1.01 | (0.87-1.17) | 0.86 | (0.75-0.99) | 0.94 | (0.82-1.06) | 0.98 | (0.78-1.24) |  |  |
|  | >6 |  | 1.13 | (0.95-1.34) | 1.02 | (0.87-1.19) | 0.37 | (0.32-0.43) | 0.78 | (0.68-0.90) | 0.93 | (0.73-1.18) |  |  |
| **Clinical T stage** | |  |  |  |  |  |  |  |  |  |  |  |  |  |
|  | T1 |  | 1.00 | (ref.) | 1.00 | (ref.) | 1.00 | (ref.) | 1.00 | (ref.) | 1.00 | (ref.) |  |  |
|  | T2 |  | 1.04 | (0.93-1.16) | 1.04 | (0.94-1.15) | 0.68 | (0.62-0.75) | 1.00 | (0.91-1.10) | 1.02 | (0.87-1.20) |  |  |
|  | T3/T4 |  | 0.68 | (0.49-0.95) | 0.88 | (0.69-1.13) | 0.28 | (0.21-0.37) | 0.74 | (0.59-0.94) | 0.93 | (0.66-1.33) |  |  |
| **Gleason score** | |  |  |  |  |  |  |  |  |  |  |  |  |  |
|  | Gleason score 6 |  | 1.00 | (ref.) | 1.00 | (ref.) | 1.00 | (ref.) | 1.00 | (ref.) | 1.00 | (ref.) |  |  |
|  | Gleason score 7 (3+4) | | 0.83 | (0.73-0.95) | 0.96 | (0.85-1.09) | 0.81 | (0.72-0.92) | 1.18 | (1.05-1.32) | 1.11 | (0.90-1.36) |  |  |
|  | Gleason score 7 (4+3) | | 0.92 | (0.79-1.08) | 1.18 | (1.02-1.36) | 0.72 | (0.63-0.83) | 0.98 | (0.86-1.12) | 1.00 | (0.80-1.26) |  |  |
|  | Gleason score 8 |  | 1.29 | (1.01-1.65) | 1.05 | (0.85-1.29) | 0.52 | (0.42-0.64) | 1.35 | (1.10-1.65) | 1.03 | (0.75-1.41) |  |  |
|  | Gleason score 9-10 |  | 1.23 | (0.89-1.69) | 1.05 | (0.81-1.35) | 0.20 | (0.15-0.26) | 1.04 | (0.82-1.32) | 0.88 | (0.62-1.26) |  |  |
| **Lymph node dissection** | | |  |  |  |  |  |  |  |  |  |  |  |  |
|  | Not performed |  | 1.00 | (ref.) | 1.00 | (ref.) | 1.00 | (ref.) | 1.00 | (ref.) | 1.00 | (ref.) |  |  |
|  | Limited |  | 0.25 | (0.14-0.43) | 0.81 | (0.48-1.38) | 1.54 | (0.89-2.66) | 1.60 | (0.92-2.78) | 1.09 | (0.43-2.73) |  |  |
|  | Extended |  | 0.12 | (0.10-0.15) | 0.66 | (0.57-0.77) | 0.85 | (0.73-0.98) | 0.79 | (0.69-0.91) | 0.50 | (0.41-0.62) |  |  |
