# Supplementary material for: Association of surgeon and hospital volume with short-term outcomes after robot-assisted radical prostatectomy: Nationwide, population-based study
Source: PLoS One. 2021 Jun 17;16(6):e0253081. doi: 10.1371/journal.pone.0253081 (PMC8211177; doi:10.1371/journal.pone.0253081)
Supplement: S1 File — (DOCX) [file pone.0253081.s003.docx]

| **List of the hospitals included** |
| --- |
| Blekingesjukhuset - Karlskrona |
| Capio S:t Görans sjukhus |
| Carlanderska sjukhuset |
| Centrallasarettet Växjö |
| Centralsjukhuset i Karlstad |
| Danderyds sjukhus |
| Falu lasarett |
| Hallands sjukhus Halmstad |
| Helsingsborgs lasarett |
| Karolinska universitetssjukhuset - Solna |
| Länssjukhuset i Kalmar |
| Länssjukhuset Ryhov |
| Norrlands universitetssjukhus Umeå |
| Sahlgrenska Universitetssjukhuset |
| Skånes universitetssjukhus - Lund |
| Skånes universitetssjukhus - Malmö |
| Södersjukhuset |
| Universitetssjukhuset i Linköping |
| Universitetssjukhuset Örebro |
| UroClinic Sophiahemmet |
| Varbergs sjukhus |
| Västmanlands sjukhus Västerås |
